# PACS2 Alleviates Sepsis‐Induced Myopathy by Activating ERK–MAPK Signalling Pathway to Suppress ER‐Phagy

**DOI:** 10.1002/jcsm.70308

**Published:** 2026-05-08

**Authors:** Xuexin Li, Zu‐an Shi, Fei He, Guo Mu, Feixiang Wang, Bowen Sun, Xiaobin Wang, Li Liu

**Affiliations:** ^1^ Department of Anesthesiology, the Fourth Affiliated Hospital Southwest Medical University Meishan Sichuan China; ^2^ Anesthesiology and Critical Care Medicine Key Laboratory of Luzhou Southwest Medical University Luzhou Sichuan Province China

**Keywords:** ERK–MAPK signalling pathway, FAM134B, MAM, PACS2, sepsis‐induced myopathy

## Abstract

**Background:**

Sepsis‐induced myopathy (SIM) is a common and life‐threatening complication, but its underlying mechanisms remain poorly understood. PACS2, a key resident protein at mitochondria‐associated endoplasmic reticulum membranes (MAMs), regulates ER homeostasis under various pathological conditions. However, whether sepsis disrupts PACS2‐dependent MAM integrity, thereby triggering ER dysfunction and muscle wasting, remains unexplored.

**Methods:**

We established a sepsis mouse model via cecal ligation and puncture (CLP) and assessed muscle function using compound muscle action potential (CMAP) recording and grip strength measurements. Muscle atrophy was evaluated by H&E staining and Western blotting. PACS2 expression was determined by Western blotting, immunohistochemistry and qRT‐PCR. MAM integrity was assessed by immunofluorescence co‐localization of IP3R and VDAC1, and ER‐phagy (reticulophagy) activation was evaluated by transmission electron microscopy, Western blotting and fluorescence microscopy. To investigate the functional role of PACS2, adeno‐associated virus (AAV)‐mediated PACS2 overexpression was performed in mouse tibialis anterior muscle and gastrocnemius muscles, followed by RNA‐sequencing analysis. The MAPK pathway proteins p‐ERK, p‐P38 and p‐JNK levels were assessed by Western blotting, and the involvement of ERK–MAPK signalling was tested pharmacologically via intraperitoneal injection of the ERK inhibitor SCH772984.

**Results:**

Septic mice developed progressive skeletal muscle atrophy (*p* < 0.001) and dysfunction (*p* < 0.01), accompanied by 56% reduction in PACS2 expression at 96 h post‐CLP (*p* < 0.01), 25% decrease in MAM integrity (*p* < 0.05) and subsequent activation of FAM134B‐mediated ER‐phagy (*p* < 0.01). AAV‐mediated PACS2 overexpression significantly alleviated muscle atrophy by restoring MAM integrity by 28% (*p* < 0.01), reducing FAM134B expression by 43% (*p* < 0.01) and attenuating ER‐phagy (*p* < 0.01). Co‐immunoprecipitation revealed no detectable direct protein–protein interaction between PACS2 and FAM134B. Transcriptome sequencing and Western blotting analysis demonstrated that PACS2 overexpression specifically activated the ERK–MAPK signalling pathway (55% increase in p‐ERK, *p* < 0.01) without affecting p‐P38 or p‐JNK levels (*p*>0.05), which suppressed FAM134B‐mediated ER‐phagy (*p* < 0.05) and ameliorated muscle atrophy (*p* < 0.05) by inhibiting nuclear translocation of TFEB (*p* < 0.01). Pharmacological ERK inhibition with SCH772984 abolished the protective effects of PACS2 by promoting TFEB nuclear translocation (*p* < 0.001) and TFEB‐mediated FAM134B expression (*p* < 0.001).

**Conclusions:**

Our findings demonstrate that SIM is closely associated with disrupted MAM integrity. PACS2 plays a critical role in maintaining MAM structural integrity and regulating FAM134B‐mediated ER‐phagy through the ERK–MAPK‐TFEB signalling axis, thereby providing novel mechanistic insights and potential therapeutic targets for SIM.

## 1 Introduction

Sepsis‐induced myopathy (SIM) is characterized by severe neuromuscular dysfunction, impaired muscle regenerative capacity and progressive muscle wasting, manifesting that substantially prolong recovery, compromise functional outcomes and increase mortality risk in critically ill patients [1]. Despite advances in sepsis management, the underlying molecular mechanisms driving SIM remain incompletely understood, limiting the development of targeted therapeutic interventions. Elucidating these pathophysiological pathways represents both a clinical imperative and a critical research priority.

Mitochondria‐associated endoplasmic reticulum membranes (MAMs) serve as specialized intracellular communication platforms that coordinate signalling processes between the endoplasmic reticulum (ER) and mitochondria. These membrane contact sites play essential regulatory roles in autophagy initiation and progression, processes fundamental to cellular homeostasis and stress adaptation [2]. Phosphofurin acidic cluster sorting protein 2 (PACS2), the first identified MAM‐resident protein, functions as a critical regulator of MAM integrity and is expressed in multiple tissues including skeletal muscle, heart, brain, liver and kidney, participating in diverse cellular processes including membrane trafficking, apoptosis and autophagy regulation [3]. Studies demonstrate that PACS2 deficiency results in fragmentation of ER and mitochondrial membrane, exacerbating renal tubular injury in diabetic mice through ER stress, ER‐phagy (reticulophagy) and mitophagy [4, 5, 6, 7]. While PACS2 has been investigated in the context of insulin resistance, cancer, neurological disorders and HIV infection, its role in skeletal muscle pathophysiology, particularly in SIM, has not been explored [8].

The ER, as the largest membrane‐bound organelle in eukaryotic cells, performs numerous physiological functions and plays a critical role in interorganellar communication [9]. ER‐phagy, the selective autophagic degradation of ER components, represents the primary quality control mechanism for maintaining ER structural and functional homeostasis. Under both physiological and pathological stress conditions, ER‐phagy prevents cellular dysfunction by clearing misfolded proteins that cannot be processed through other ER quality control pathways [10]. FAM134B, the first identified ER‐phagy receptor, regulates ER‐phagy and ER remodelling through two critical functional domains: the LC3‐interacting region (LIR), which recruits and binds autophagy‐related LC3/GABARAP proteins, and the reticulon homology domain (RHD), which induces ER membrane curvature [11]. Both domains mediate the sequestration of ER fragments into autophagosomes for subsequent lysosomal degradation [12]. Recent evidence has implicated FAM134B in myogenic regulation [13, 14]. However, its potential involvement in SIM pathogenesis remains unknown.

This study aimed to investigate the underlying mechanism of SIM, specifically focusing on PACS2‐mediated MAM integrity alterations and the involvement of ER‐phagy. Furthermore, we sought to explore the interaction between PACS2 and ER‐phagy receptor FAM134B and to clarify the pathogenic mechanisms of SIM through targeted interventions.

## 2 Methods

### 2.1 Animals

Male C57BL/6J mice (6–8 weeks old) obtained from the Laboratory Animal Center of Southwest Medical University were housed in our facility for 2 weeks before experimentation to allow for acclimatization. The animals were maintained at 20°C–25°C with 40%–70% relative humidity under a 12‐h light/dark cycle, with food and water provided ad libitum. Thirty‐two specific pathogen‐free (SPF) male C57BL/6J mice were randomly allocated into a Sham group (*n* = 12) and cecal ligation and puncture (CLP) group (*n* = 20). The Sham group was further subdivided into Sham‐24 h (*n* = 6) and Sham‐96 h (*n* = 6) subgroups, while the CLP group was subdivided into CLP‐24 h (*n* = 10) and CLP‐96 h (*n* = 10) subgroups. All animal experimental protocols were reviewed and approved by the Animal Ethics Committee of Southwest Medical University on November 4, 2024 (approval number: SWMU20240075).

### 2.2 Animal Model of Sepsis

The animals were subjected to either CLP surgery to establish the sepsis model or sham surgery (laparotomy without CLP) as a control. Briefly, mice were anesthetized with sodium pentobarbital (1%, 50 mg/kg intraperitoneally [i.p.]) and received buprenorphine (0.05 mg/kg i.p.) for analgesia. A small paramedian incision approximately 1 cm in length was made through the skin and abdominal wall. The cecum was located and externalized using forceps. For the CLP procedure, the distal half of the cecum was ligated with a single knot using a 4.0 silk suture and perforated once with a 20‐gauge needle to induce moderate‐grade sepsis. A small amount of faecal material was extruded through the puncture site by gently squeezing the cecum. The cecum was then returned to the peritoneal cavity, and the abdominal incision was closed with 4.0 silk sutures. The sham group underwent identical procedures except for CLP. Both groups received postoperative supportive care, including fluid resuscitation (50 mL/kg of 0.9% NaCl, subcutaneously [s.c.]) and antibiotic prophylaxis (30 mg/kg ceftriaxone and clindamycin, s.c.) immediately following the CLP and non‐CLP procedures.

### 2.3 Haematoxylin and Eosin Staining

Muscle samples were fixed in 4% paraformaldehyde for 24 h and subsequently embedded in paraffin. Transverse serial sections (5 μm thick) were cut from the mid‐belly region of each sample using a microtome (Leica, Germany) and mounted on glass slides. Following deparaffinization and rehydration, sections were stained with haematoxylin and eosin (H&E) according to standard protocols. Myofiber cross‐sectional area (CSA) was measured from H&E‐stained sections using Image‐Pro Plus 6.0 (Media Cybernetics, USA). To account for overall muscle atrophy, CSA was normalized to the corresponding muscle wet weight and expressed as μm^2^/mg.

### 2.4 Plasmid Construction

All plasmids used in this study were constructed by Hanbio Biotechnology Co. Ltd. (Shanghai, China). Specific primers were designed based on the mouse PACS2 gene sequence, and the target gene fragment was amplified by polymerase chain reaction (PCR) using high‐fidelity polymerase. The PCR product was purified and verified by agarose gel electrophoresis. Concurrently, the adeno‐associated virus (AAV) vector backbone was linearized using appropriate restriction endonucleases. The purified PCR fragment was then recombined with the linearized vector using HB infusion one‐step cloning technology. The recombination product was transformed into DH5α competent cells, and positive clones were screened by colony PCR following plate culture. Positive clones were verified by Sanger sequencing to confirm sequence accuracy. Finally, plasmid DNA was extracted from validated clones and subjected to concentration determination, purity analysis and sequencing verification to ensure that the plasmid quality met the requirements for viral packaging.

### 2.5 Immunohistochemistry Staining

Skeletal muscle samples were fixed and embedded in paraffin and sectioned at 4‐μm thickness. The sections were then deparaffinized and rehydrated through xylene, absolute ethanol, 95% alcohol, 90% alcohol and 80% alcohol, respectively. Antigen retrieval was performed, followed by blocking of endogenous peroxidase activity and blocking with 5% goat serum for 1 h at room temperature. Sections were then incubated with primary antibody against PACS2 (Proteintech, 19 508‐1‐AP, 1:100) overnight at 4°C. After washing three times with PBS, sections were incubated with secondary antibody for 30 min at room temperature. Then, the nuclei were counterstained with haematoxylin. After sealing, the images were acquired using a Digital Pathology Slide Scanner (KFBIO, KF‐PRO‐002).

### 2.6 AAV Injection Into Skeletal Muscle of Mice

For AAV local injections into muscle, male C57BL/6J mice (6–8 weeks old) were randomized into AAV‐CON‐Sham and AAV‐PACS2‐Sham groups (*n* = 10), and AAV‐CON‐CLP and AAV‐PACS2‐CLP groups (*n* = 10). Prior to AAV administration, mice were anesthetized by isoflurane inhalation. For intramuscular injection, the right TA and GAS muscles of mice were injected with 8 and 10 μL of AAV‐PACS2 in PBS, respectively, while the left TA and GAS muscles of mice were injected with 8 and 10 μL of AAV‐CON in PBS, respectively. All muscle injections are performed using a three‐point injection technique. CLP modelling was conducted 4 weeks after AAV injection.

### 2.7 ERK Inhibitor Injection

Male C57BL/6J mice were randomly assigned to the following groups: (1) Sham group, (2) CLP group, (3) CLP+AAV‐PACS2 group, (4) CLP+SCH772984 group and (5) CLP+AAV‐PACS2+SCH772984 group. SCH772984 (Selleckchem, S7101) was dissolved in DMSO to prepare a stock solution at 25 mg/mL. The working solution was prepared by diluting the stock in a vehicle consisting of 4% DMSO, 40% PEG300 (Selleckchem, S6704), 5% Tween‐80 (Selleckchem, S6702) and 51% ddH_2_O, according to the manufacturer's recommendations. Mice in the SCH772984‐treated groups received SCH772984 at 10 mg/kg body weight via intraperitoneal injection twice daily for 4 days following CLP surgery until the termination of the experiment. Control mice received an equivalent volume of vehicle via the same route and schedule.

### 2.8 Immunoblotting

Skeletal muscle samples were lysed on ice for 30 min with cold RIPA buffer supplemented with PMSF. For detection of phosphorylated proteins, phosphatase inhibitors were additionally included in the lysis buffer. Lysates were centrifuged at 12000 g for 15 min at 4°C, and the supernatants were collected. Protein concentrations were quantified using a BCA protein assay kit (Beyotime, P0010). Equal amounts of protein (20 μg per lane) were separated by 10% or 12.5% SDS‐PAGE and then transferred to PVDF membranes. The membranes were blocked with 5% nonfat milk in TBST for 2 h at room temperature, followed by incubation with primary antibodies overnight at 4°C. After three washes with TBST, the membranes were incubated with HRP‐conjugated secondary antibodies for 1 h at room temperature. Following additional washes, protein bands were visualized using an ECL detection system and quantified with ImageJ software. Primary antibodies and dilutions were as follows: anti‐MuRF1 (sc‐398 608, 1:1000), anti‐Atrogin‐1 (sc‐166 806, 1:1000) and anti‐LC3β (sc‐271 625, 1:1000) from Santa Cruz Biotechnology; anti‐TFEB (ab245350, 1:2000) from Abcam; anti‐PACS2 (19 508‐1‐AP, 1:1000), anti‐FAM134B (21 537‐1‐AP, 1:1000), anti‐GAPDH (60 004‐1‐Ig, 1:15000), anti‐Tubulin (66 031‐1‐Ig, 1:20000), anti‐β‐actin (66009‐1‐Ig, 1:20000), anti‐Lamin B1 (12987‐1‐AP, 1:10000), anti‐p62 (66184‐1‐Ig, 1:2000), anti‐phospho‐P38 (Thr180/Tyr182, 28 796–1‐AP, 1:1000) and anti‐P38 (14064‐1‐AP, 1:2000) from Proteintech; anti‐phospho‐ERK (Thr202/Tyr204 and Thr185/Tyr187, ET1610‐13, 1:1000) and anti‐ERK (ET1601‐29, 1:1000) from HUABIO Biotechnology; and anti‐phospho‐JNK (Thr183/Tyr185, P20363, 1:1000) and anti‐JNK (30 369, 1:1000) from ProMab Biotechnology.

### 2.9 Co‐Immunoprecipitation (Co‐IP) Assay

Co‐IP was performed using a commercial kit (Beyotime, P2179S). Muscle tissues were homogenized and lysed on ice in IP lysis buffer containing protease inhibitors. Following 30‐min incubation and centrifugation, protein concentration in the supernatant was measured with a BCA kit (Beyotime, P0010). Antibodies were pre‐incubated with protein A/G magnetic beads for 1 h at room temperature, then mixed with 800 μg of lysate and rotated overnight at 4°C. After three washes, the immunoprecipitated complexes were eluted by boiling in SDS‐PAGE loading buffer and analysed by Western blot. To examine the PACS2‐FAM134B interaction, anti‐PACS2 antibody (Proteintech, 19 508‐1‐AP) was used for immunoprecipitation and the blot was probed with anti‐FAM134B (Proteintech, 21 537‐1‐AP). To validate the IP3R‐VDAC1 interaction, anti‐IP3R antibody (Santa Cruz, SC‐377518) was used for immunoprecipitation, followed by immunoblotting with anti‐VDAC1 (Abcam, ab14734).

### 2.10 Nuclear and Cytoplasmic Fractions

Nuclear and cytoplasmic fractions were isolated from mouse skeletal muscle tissue using the Nuclear and Cytoplasmic Protein Extraction Kit (Thermo Scientific, 78 833) according to the manufacturer's instructions. Briefly, approximately 50 mg of tissue was rinsed with ice‐cold PBS, homogenized in lysis buffer containing protease inhibitors, and centrifuged to collect intact cells. The cells were then subjected to low‐osmotic treatment and detergent‐based separation to sequentially obtain cytoplasmic and nuclear fractions. The efficiency of fractionation was verified by Western blot analysis using β‐actin as a cytoplasmic marker and Lamin B1 as a nuclear marker. Only samples showing clear enrichment of β‐actin in the cytoplasmic fraction and Lamin B1 in the nuclear fraction, with minimal cross‐contamination, were used for subsequent experiments. Thereafter, the nuclear protein extracts were subjected to Western blot to detect the protein level of TFEB.

### 2.11 Quantification and Statistical Analysis

All data are expressed as means ± SD from at least three independent experiments unless otherwise stated. Statistical analyses were performed using GraphPad Prism version 10 (GraphPad, La Jolla, CA, USA). Normality of data distribution was assessed using the Shapiro–Wilk test. For comparisons between two groups, an unpaired Student's *t*‐test (two‐tailed) was used for normally distributed data. For comparisons among three or more groups, one‐way analysis of variance (ANOVA) followed by Tukey's post hoc test was employed. Statistical significance was defined as *p* < 0.05. In all figures, significance levels are indicated as follows: *p < 0.05, **p < 0.01, ***p < 0.001, and ****p < 0.0001. Each data point and the number of biological replicates (*n*) are specified in the corresponding figure legends.

Additional experimental details are provided in the Supporting Information.

## 3 Results

### 3.1 Sepsis‐Induced Skeletal Muscle Atrophy and Weakness in Mice

**FIGURE 1:**
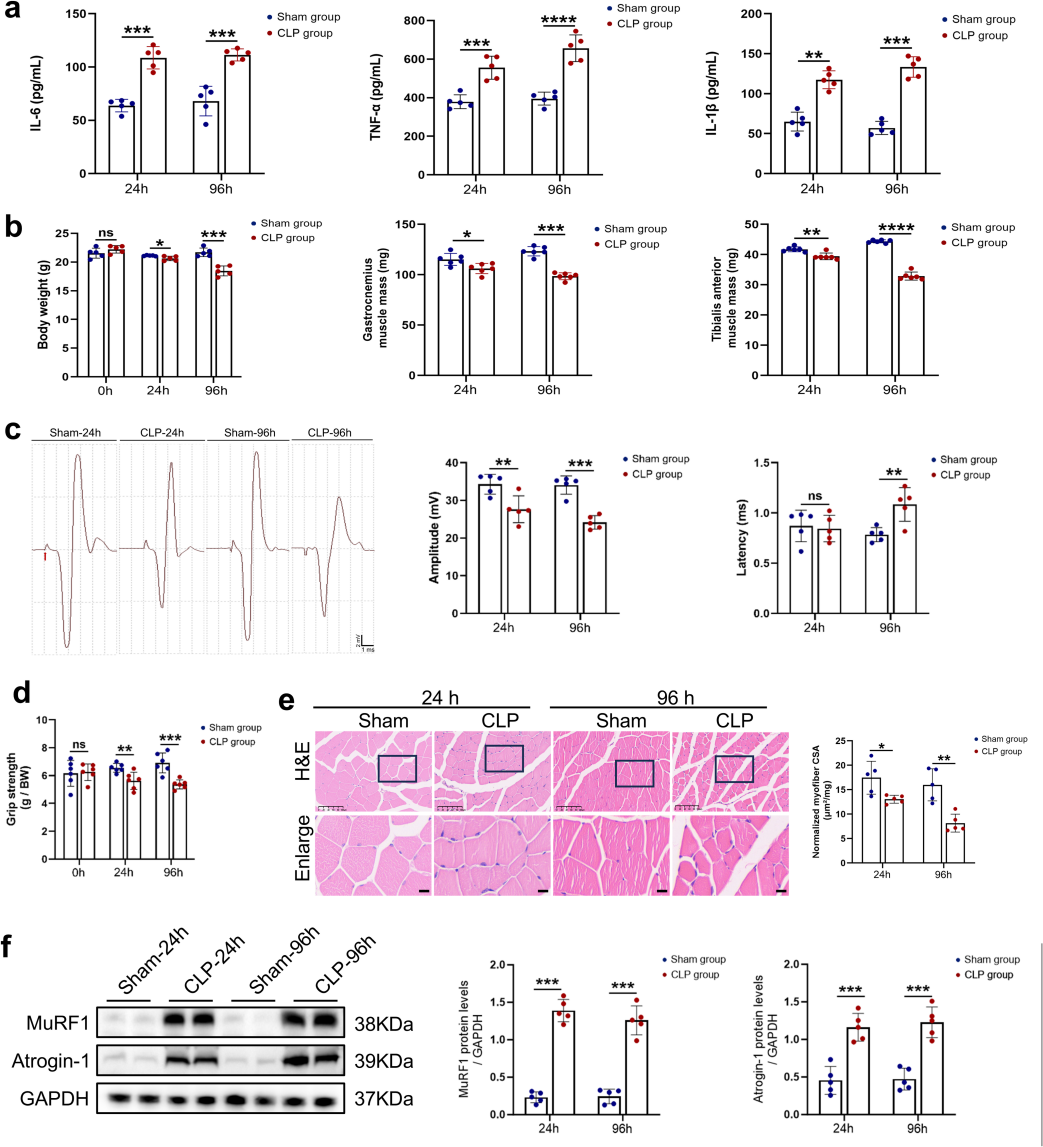
Sepsis‐induced skeletal muscle atrophy and weakness in mice. Sepsis mice model was established by CLP. Sham‐24 h and sham‐96 h groups were only sham‐operated; CLP‐24 h and CLP‐96 h groups were detected at 24‐ and 96‐h after operation. Blue dots represent sham group, and red dots represent CLP group. Comparisons: CLP‐24 h vs. Sham‐24 h and CLP‐96 h vs. Sham‐96 h. (a) IL‐6, TNF‐α, IL‐1β were detected in serum of mice (*n* = 5). (b) Body weight of mice (*n* = 5) and wet weight of gastrocnemius (GAS) muscle and tibialis anterior (TA) muscle (*n* = 6). (c) Skeletal muscle function was measured by measuring sciatic nerve GAS muscle Compound muscle action potential (CMAP). Red arrow indicates single stimulation. Amplitude was determined from the maximum negative peak to the maximum positive peak of the biphasic wave, which indicates the number of depolarizing muscle fibres (*n* = 5). Latency was measured from stimulation onset to CMAP response initiation, which reflects nerve‐to‐muscle signal conduction time (*n* = 5). (d) Relative grip strength was represented by measuring the ratio of the maximum grip strength of the forelimb of mice to their own body weight (*n* = 6). (e) Quantification of GAS muscle fibre cross‐sectional areas (CSA) from HE‐stained sections (*n* = 5). Representative images at 20 × magnification are shown (scale bar: 100 μm). CSA values were normalized to muscle wet weight and expressed as μm^2^/mg. (f) Western blot analysis of the dynamic changes of MuRF1 and Atrogin‐1 protein levels in TA muscle of each group, results normalized to levels of GAPDH (*n* = 5). Data are means ± SD *P* values determined through Students t test. *ns*, not significant; *p < 0.05; **p < 0.01; ***p < 0.001; ****p < 0.0001.

To investigate the impact of sepsis on skeletal muscle, a murine sepsis model was established via CLP. Serum levels of the pro‐inflammatory cytokines IL‐6, TNF‐α and IL‐1β were significantly increased in septic mice compared to sham‐operated controls (Figure 1a), confirming successful induction of the sepsis model. Subsequent analysis demonstrated that sepsis resulted in significant decreases in both body weight and muscle mass, with GAS and TA muscle weights reduced by approximately 20% and 25%, respectively, at 96 h post‐CLP (Figure 1b).

To assess the functional consequences of sepsis on skeletal muscle, neuromuscular function was evaluated using CMAP recordings. Septic mice exhibited decreased CMAP amplitude and prolonged latency (Figure 1c), indicating impaired neuromuscular transmission. Consistent with these electrophysiological findings, grip strength testing revealed significantly diminished forelimb strength in septic mice (reduced by 23% at 96 h post‐CLP; Figure 1d). Histological examination via H&E staining of GAS muscles demonstrated that normalized myofiber CSA was significantly decreased by up to 50% at 96 h post‐CLP (Figure 1e).

Moreover, Western blot analysis of TA muscle tissue revealed significantly increased expression of the muscle‐specific E3 ubiquitin ligases MuRF1 and Atrogin‐1, which are established markers of muscle protein degradation (Figure 1f). Collectively, these findings demonstrate that CLP‐induced sepsis successfully recapitulates the key features of sepsis‐associated skeletal muscle atrophy and functional impairment in mice.

### 3.2 Sepsis Leads to FAM134B‐Mediated Activation of ER‐Phagy in Mouse Skeletal Muscle

**FIGURE 2:**
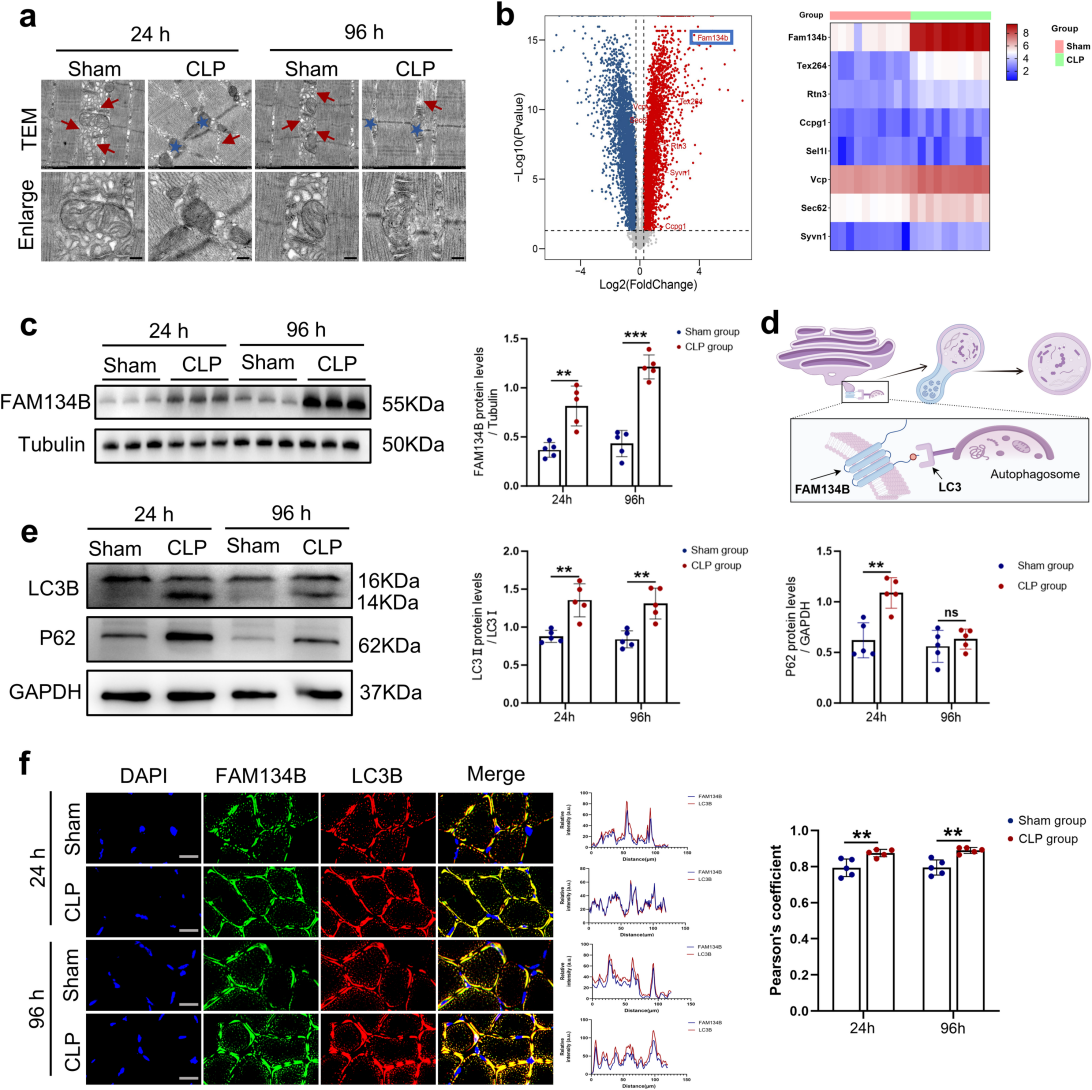
Sepsis leads to FAM134B‐mediated activation of ER‐phagy in mouse skeletal muscle. Mice were divided into Sham‐24 h, CLP‐24 h, Sham‐96 h and CLP‐96 h groups. Comparisons: CLP‐24 h vs. Sham‐24 h and CLP‐96 h vs. Sham‐96 h. (a) Observation of ER ultrastructural and quantity by TEM. Red arrows indicate ER structures and blue stars indicate the disrupted mitochondria. Scale bars: 500 nm. (b) Transcriptome sequencing analysis of TA muscle from Sham‐96 h and CLP‐96 h group mice (*n* = 10), volcano map and heat map showing differentially expressed genes. (c) Western blot analysis of the dynamic changes of FAM134B protein levels in TA muscle of each group, results normalized to levels of Tubulin (*n* = 5). (d) Schematic diagram of FAM134B binding to the autophagy marker LC3 during ER‐phagy. (e) Western blot analysis of the dynamic changes of LC3B and p62 protein levels in TA muscle of each group, LC3B results normalized to LC3II/I, p62 results normalized to levels of GAPDH (*n* = 5). (f) Immunofluorescence analysis of TA muscle. Blue fluorescence represents DAPI; green fluorescence represents FAM134B; red fluorescence represents LC3B. Merged images show co‐localization of FAM134B and LC3B, indicating ER‐phagy activity (*n* = 5). Data are means ± SD *P* values determined through Students *t*‐test. *ns*, not significant; **p < 0.01; ***p < 0.001.

Given the severe muscle atrophy observed in septic mice, we performed electron microscopy analysis of the TA muscle to examine ultrastructural changes at the subcellular level. The results revealed that sepsis induced marked structural damage to the ER, characterized by reduced ER content and disrupted ER morphology. Concomitantly, mitochondrial integrity was severely compromised, characterized by disrupted mitochondrial membranes and disorganized cristae (Figure 2a), which is consistent with previous reports implicating mitochondrial dysfunction in SIM pathogenesis [15, 16].

To investigate whether ER‐phagy contributes to SIM development, we performed RNA‐sequencing (RNA‐seq) analysis of TA muscle from septic mice. Volcano plots and heatmaps demonstrated upregulation of multiple ER‐phagy‐related genes, including FAM134B, TEX264, SEC62, RTN3 and CCPG1. Notably, FAM134B exhibited the most pronounced increase, with approximately fourfold upregulation (Figure 2b). We subsequently validated FAM134B protein expression by Western blot analysis, which revealed that FAM134B levels were increased at 24‐h post‐CLP and remained elevated until 96 h, with approximately threefold induction (Figure 2c).

Given that LC3 mediates ER‐phagy via its interaction with the LIR motif of FAM134B (Figure 2d), we next examined the expression levels of LC3. Western blot analysis demonstrated that sepsis increased the expression of both LC3 and p62 (Figure 2e). Furthermore, immunofluorescence staining of the TA muscle demonstrated enhanced co‐localization of FAM134B and LC3B in septic mice (Figure 2f). Collectively, these findings indicate that sepsis triggers the activation of FAM134B‐mediated ER‐phagy in mouse skeletal muscle.

### 3.3 Sepsis Leads to Decreased PACS2 Expression and Structural Damage of MAM

**FIGURE 3:**
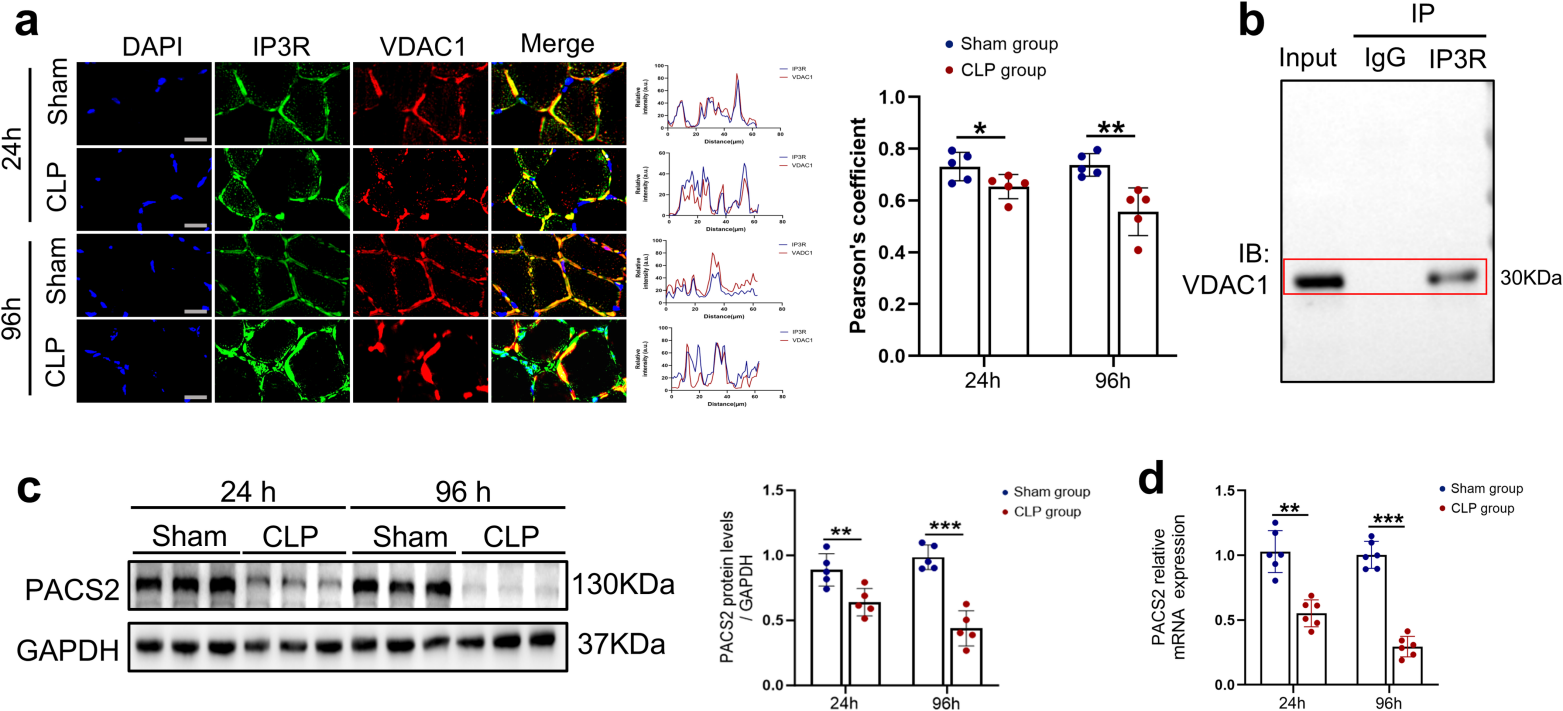
Sepsis leads to decreased PACS2 expression and structural damage of MAM. Mice were divided into Sham‐24 h, CLP‐24 h, Sham‐96 h and CLP‐96 h groups. Comparisons: CLP‐24 h vs. Sham‐24 h and CLP‐96 h vs. Sham‐96 h. (a) Immunofluorescence analysis of TA muscle in each group, blue fluorescence represents DAPI, green fluorescence represents IP3R, red fluorescence represents VDAC1, and the merged images indicated co‐localization of IP3R and VDAC1 represents the integrity of MAM structure (*n* = 5). (b) Co‐immunoprecipitation (Co‐IP) analysis demonstrates detectable interaction between IP3R and VDAC1. (c) Western blot analysis of the dynamic changes of PACS2 protein levels in TA muscle of each group, results normalized to levels of GAPDH (*n* = 5). (d) qRT‐PCR analysis of PACS2 mRNA expression in TA muscle of each group (*n* = 6). Data are means ± SD *P* values determined through Students t test. *p < 0.05; **p < 0.01; ***p < 0.001.

Given that the ER physically and functionally interacts with mitochondria at MAM contact sites and that MAM integrity is critical for ER homeostasis, disruption of MAM structure can induce ER‐phagy [17]. To determine whether the enhanced ER‐phagy was associated with MAM structural disruption, we assessed MAM integrity by examining the co‐localization of IP3R and VDAC1, two key MAM‐resident proteins that physically interact at the ER‐mitochondria contact sites, in skeletal muscles from septic mice. Immunofluorescence analysis revealed that sepsis significantly decreased IP3R and VDAC1 co‐localization (reduced by 25% at 96 h post‐CLP), indicating MAM structural disruption (Figure 3a). Given the similar staining patterns observed for IP3R and VDAC1, we performed Co‐IP assays to validate their physical interaction, and the results confirmed the interaction between these two proteins (Figure 3b).

PACS2, a key regulatory protein of MAM dynamics, plays a crucial role in maintaining the ER‐mitochondrial tethering and ER homeostasis. Recent studies have shown that decreased PACS2 expression exacerbates renal tubular injury in diabetic mice by promoting ER stress and autophagy [7, 18]. We therefore hypothesized that sepsis‐induced MAM disruption and subsequent ER‐phagy activation might be linked to downregulation of PACS2. To test this hypothesis, we first assessed PACS2 protein expression by Western blot analysis. Results revealed that PACS2 expression was significantly decreased at 24 h post‐CLP (approximately 28%) and remained suppressed until 96 h (approximately 56%) (Figure 3c).

Consistent with these findings, immunohistochemical staining (Figure S1) and qRT‐PCR analysis (Figure 3d) confirmed the downregulation of PACS2 at both the protein and mRNA levels. Collectively, these data suggest that sepsis‐induced ER‐phagy activation is associated with MAM structural disruption resulting from PACS2 downregulation.

### 3.4 PACS2 Overexpression Alleviated Skeletal Muscle Atrophy in Septic Mice

**FIGURE 4:**
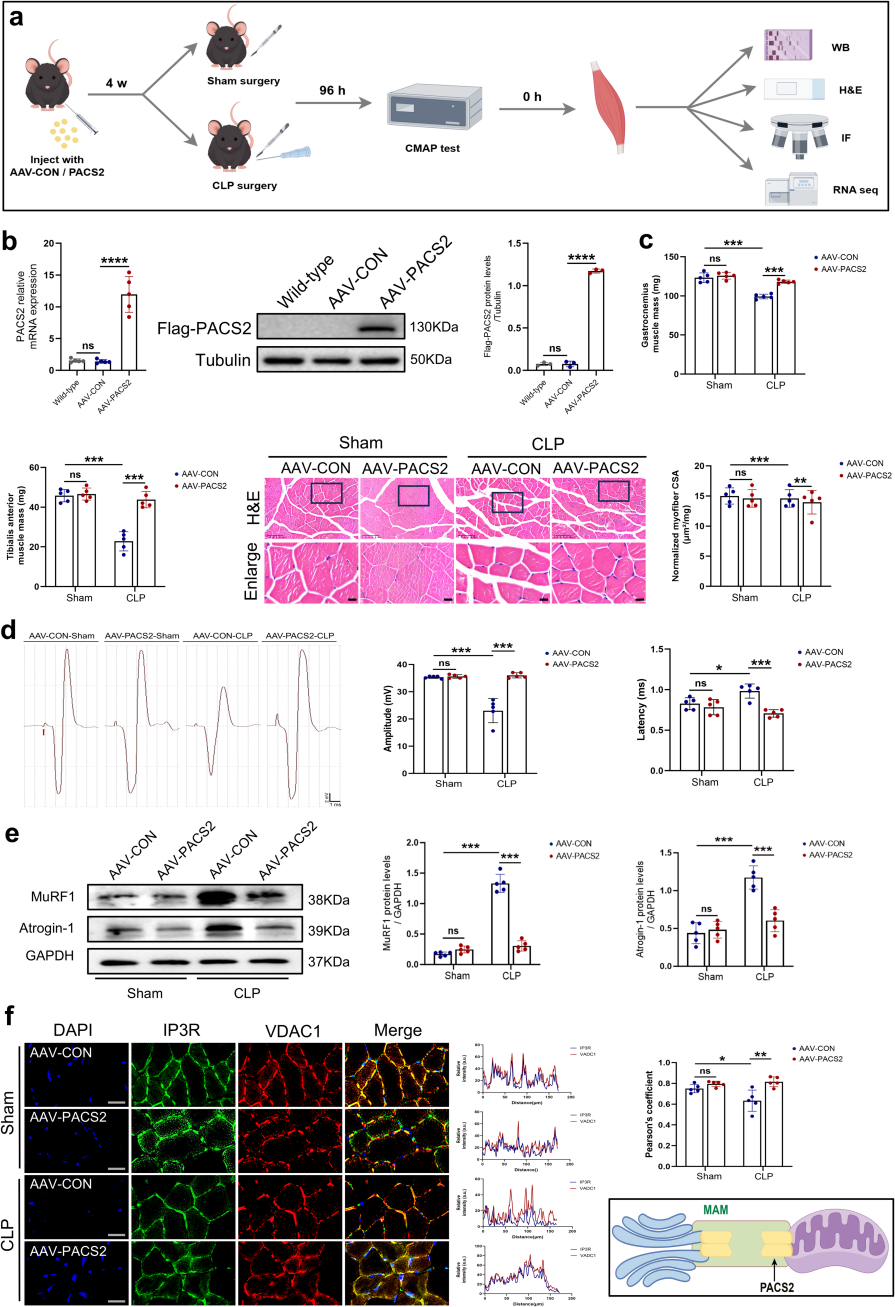
PACS2 overexpression alleviated skeletal muscle atrophy in septic mice. Mice received AAV‐CON or AAV‐PACS2 for PACS2 overexpression, followed by CLP or sham operation, and were divided into AAV‐CON‐Sham, AAV‐PACS2‐Sham, AAV‐CON‐CLP and AAV‐PACS2‐CLP groups. (a) Schematic diagram of the experimental timeline showing AAV administration, CLP modelling, and subsequent assessments. (b) qRT‐PCR (*n* = 5) and Western blot (*n* = 3) analysis of PACS2 expression in TA muscle following AAV‐CON or AAV‐PACS2 intervention, with results normalized to levels of Tubulin. (c) Wet weight of GAS muscle and TA muscle isolated (*n* = 5). And quantification of GAS muscle fibre CSA from HE‐stained sections (*n* = 5). Representative images at 20 × magnification are shown (scale bar: 100 μm). CSA values were normalized to muscle wet weight and expressed as μm^2^/mg. (d) Skeletal muscle function was measured by measuring sciatic nerve GAS muscle CMAP. Red arrow indicates single stimulation. Amplitude was determined from the maximum negative peak to the maximum positive peak of the biphasic wave, which indicates the number of depolarizing muscle fibres (*n* = 5). Latency was measured from stimulation onset to CMAP response initiation, which reflects nerve‐to‐muscle signal conduction time (*n* = 5). (e) Western blot analysis of the dynamic changes of MuRF1 and Atrogin‐1 protein levels in TA muscle of each group, results normalized to levels of GAPDH (*n* = 5). (f) Immunofluorescence analysis of TA muscle in each group, blue fluorescence represents DAPI, green fluorescence represents IP3R, red fluorescence represents VDAC1, and the merged images indicated co‐localization of IP3R and VDAC1 represents the integrity of MAM structure (*n* = 5). And Schematic diagram illustrates PACS2 localization and its role in regulating MAM structural stability. Data are means ± SD *P*‐value determined by ordinary one‐way ANOVA. *ns*, not significant; *p < 0.05; **p < 0.01; ***p < 0.001; ****p < 0.0001.

To investigate whether PACS2 downregulation contributes to skeletal muscle atrophy in septic mice, we delivered AAV vectors encoding PACS2 via direct intramuscular injection into the TA and GAS muscles 4 weeks prior to CLP surgery (Figure 4a). Consistent with qRT‐PCR data, Western blot analysis confirmed approximately 12‐fold overexpression of PACS2 in the skeletal muscle of mice (Figure 4b).

To evaluate the protective effect of PACS2 overexpression on muscle injury in septic mice, we first examined the effect of PACS2 overexpression on sepsis‐induced muscle mass loss. The results showed that PACS2 overexpression prevented the reduction in GAS and TA muscle mass, and H&E staining of the GAS muscle revealed that sepsis induced a significant reduction in normalized myofiber CSA, which was markedly attenuated by PACS2 overexpression (Figure 4c). We then assessed muscle function using CMAP recordings. Results showed that sepsis reduced CMAP amplitude and prolonged latency, whereas PACS2 overexpression attenuated these electrophysiological deficits (Figure 4d), indicating improved neuromuscular function. Consistent with these histological findings, PACS2 overexpression reduced sepsis‐induced upregulation of MuRF1 and Atrogin‐1 by approximately 77% and 50%, respectively (Figure 4e).

Next, we assessed the impact of PACS2 overexpression on MAM structure. Immunofluorescence staining showed that sepsis reduced the co‐localization of IP3R and VDAC1, whereas PACS2 overexpression restored MAM structure in the skeletal muscle of septic mice, increasing co‐localization by approximately 28% (Figure 4f). These findings collectively demonstrate that PACS2, as a resident protein localized at MAM, maintains the ER‐mitochondria contact site integrity and thereby protects against sepsis‐induced skeletal muscle atrophy.

### 3.5 PACS2 Overexpression Suppresses FAM134B‐Mediated ER‐Phagy in Septic Skeletal Muscle

**FIGURE 5:**
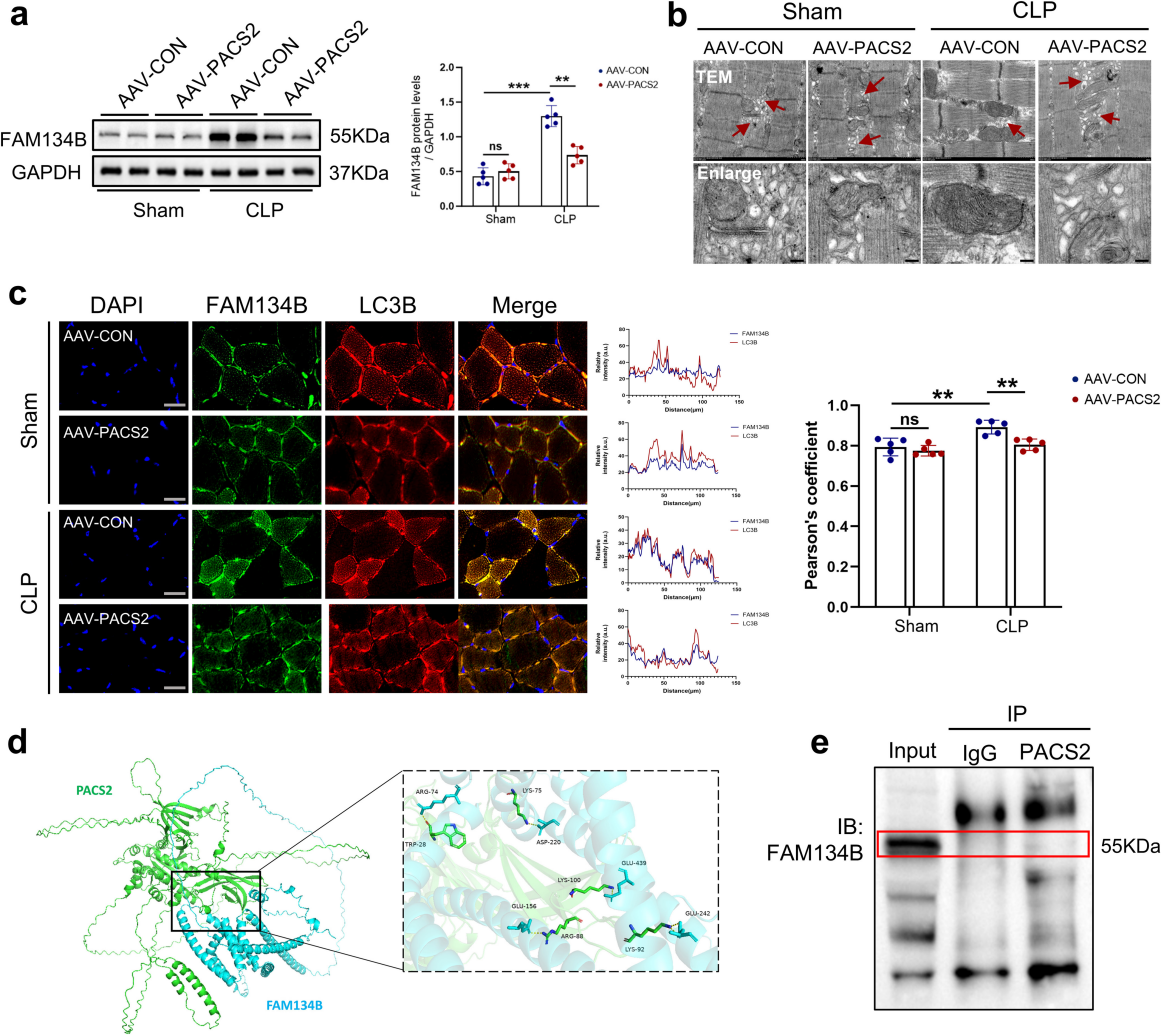
PACS2 overexpression suppresses FAM134B‐mediated ER‐phagy in septic skeletal muscle. Mice were divided into AAV‐CON‐Sham, AAV‐PACS2‐Sham, AAV‐CON‐CLP and AAV‐PACS2‐CLP groups. (a) Western blot analysis of the dynamic changes of FAM134B protein levels in TA muscle of each group, results normalized to levels of GAPDH (*n* = 5). (b) Observation of ER ultrastructural and quantity by TEM. Red arrows indicated ER structure. Scale bars: 500 nm. (c) Immunofluorescence analysis of TA muscle. Blue fluorescence represents DAPI; green fluorescence represents FAM134B; red fluorescence represents LC3B. Merged images show co‐localization of FAM134B and LC3B, indicating ER‐phagy activity (*n* = 5). (d) Protein–protein interaction between PACS2 and FAM134B predicted using the STRING database. (e) Co‐immunoprecipitation (Co‐IP) analysis demonstrates no detectable physical interaction between PACS2 and FAM134B. Data are means ± SD *P*‐value determined by ordinary one‐way ANOVA. *ns*, not significant; **p < 0.01; ***p < 0.001.

To investigate whether the protective effect of PACS2 overexpression against skeletal muscle atrophy in septic mice is mediated through suppression of ER‐phagy, we assessed the expression of the ER‐phagy receptor FAM134B. Western blot analysis showed that PACS2 overexpression significantly reduced FAM134B expression by approximately 43% in septic mice (Figure 5a). To directly visualize the impact of PACS2 overexpression on ER ultrastructure in the skeletal muscle of septic mice, we performed transmission electron microscopy on the TA muscle. Ultrastructural analysis demonstrated that sepsis resulted in decreased ER abundance and disrupted ER architecture. In contrast, PACS2 overexpression substantially attenuated this damage, restoring ER structural integrity and increasing ER content (Figure 5b). Correspondingly, immunofluorescence analysis demonstrated reduced co‐localization of FAM134B and LC3B in PACS2‐overexpressing mice, indicating that PACS2 overexpression suppressed ER‐phagy in skeletal muscle of septic mice (Figure 5c).

Given that the RHD domain of FAM134B mediates interactions with diverse proteins via protein clustering [13], we next explored whether PACS2 interacts with FAM134B directly. We first utilized the STRING database to predict potential protein–protein interactions between PACS2 and FAM134B. In silico analysis predicted potential interaction sites between PACS2 and FAM134B (Figure 5d). However Co‐IP experiments failed to detect physical interaction between the two proteins (Figure 5e). Considering that PACS2 overexpression in the skeletal muscle of septic mice led to a decreased expression of FAM134B, but no detectable direct binding was observed, we hypothesized that PACS2 may regulate FAM134B expression through an indirect mechanism.

### 3.6 PACS2 Overexpression Suppresses FAM134B Expression via Activation of the ERK–MAPK Signalling Pathway

**FIGURE 6:**
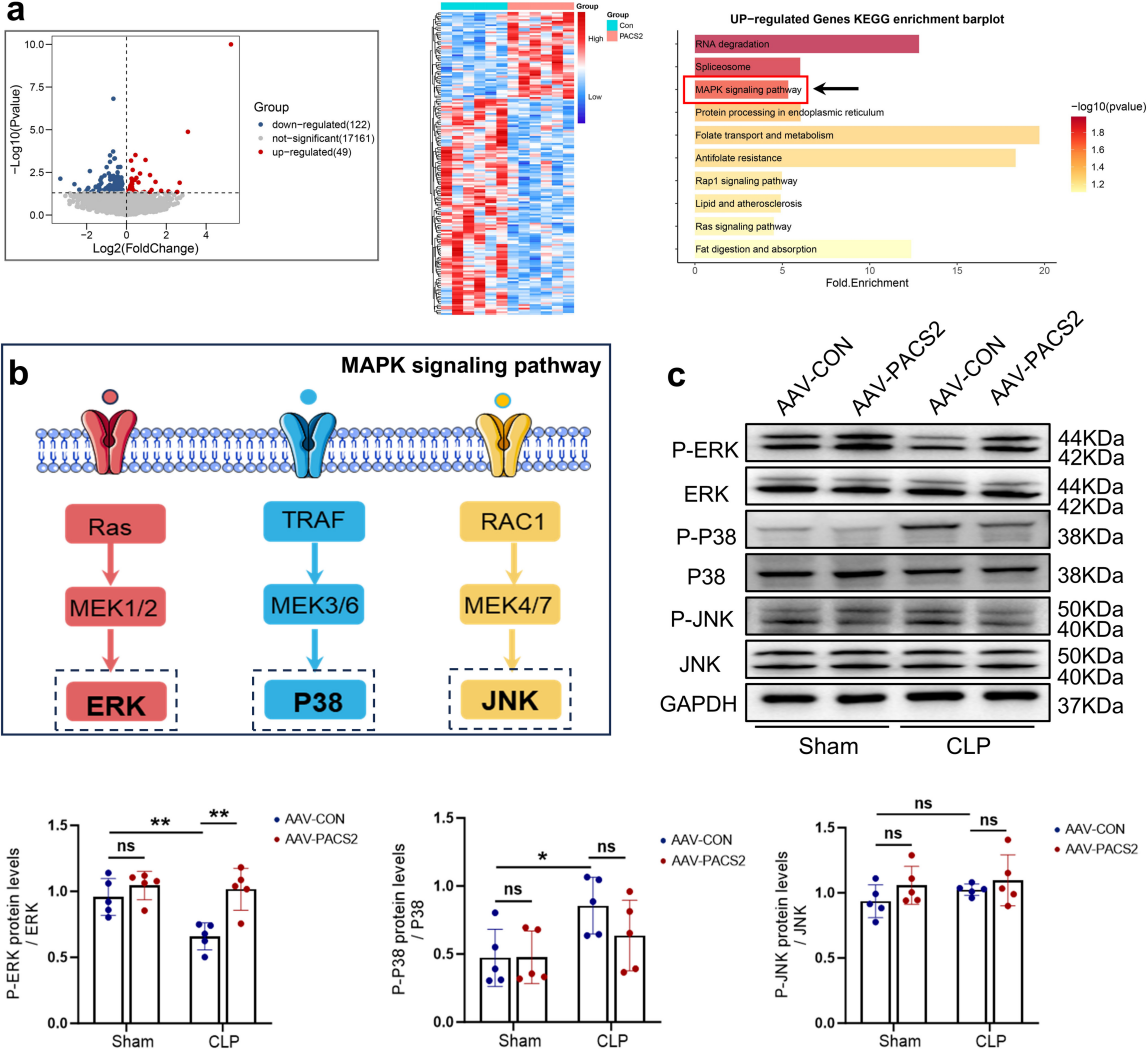
PACS2 overexpression reduces FAM134B‐mediated ER‐phagy by activating ERK–MAPK signalling pathway. (a) Transcriptome sequencing analysis of TA muscle from mice in the PACS2‐overexpressing and control groups after CLP modelling. Volcano plot and heatmap showing differentially expressed genes (*n* = 6). Kyoto Encyclopedia of Genes and Genomes (KEGG) pathway enrichment analysis of differentially expressed genes from the transcriptome data. (b) Schematic diagram illustrating the three major MAPK signalling cascades (ERK, P38, and JNK) and their downstream pathways. (c) Western blot analysis of ERK, P38 and JNK total protein levels and phosphorylated levels in TA muscle from AAV‐CON‐Sham, AAV‐PACS2‐Sham, AAV‐CON‐CLP and AAV‐PACS2‐CLP groups. Results are expressed as phosphorylation ratios: p‐ERK/ERK, p‐P38/P38 and p‐JNK/JNK (*n* = 5). Data are means ± SD *P*‐value determined by ordinary one‐way ANOVA. *ns*, not significant; *p < 0.05; **p < 0.01.

To elucidate the underlying mechanism, we performed RNA‐seq analysis on TA muscles from septic mice treated with either empty vector (AAV‐CON) or PACS2‐overexpressing AAV (AAV‐PACS2). RNA‐seq revealed that PACS2 overexpression resulted in 49 upregulated and 122 downregulated genes in septic mouse skeletal muscle. KEGG pathway enrichment analysis of the upregulated genes identified significant activation of the MAPK signalling pathway (Figure 6a). Based on the sequencing results, we examined the expression of three signature proteins of the MAPK pathway: ERK, P38, JNK and their phosphorylated levels (Figure 6b). The results showed that sepsis decreased p‐ERK levels and increased p‐P38 levels but had no effects on p‐JNK levels. Importantly, PACS2 overexpression significantly increased p‐ERK levels by approximately 55% in skeletal muscle of septic mice, while having no effect on p‐P38 levels (Figure 6c). The ERK–MAPK signalling pathway plays critical roles in multiple biological processes, including metabolism, transcription and cell proliferation [19]. Collectively, these findings suggest that PACS2 overexpression may alleviate skeletal muscle atrophy in septic mice by activating the ERK–MAPK signalling pathway, thereby suppressing FAM134B‐mediated ER‐phagy activation.

### 3.7 ERK Activation Reduces the Transcriptional Levels of FAM134B in the Skeletal Muscles of Septic Mice by Inhibiting the Nuclear Translocation of TFEB

**FIGURE 7:**
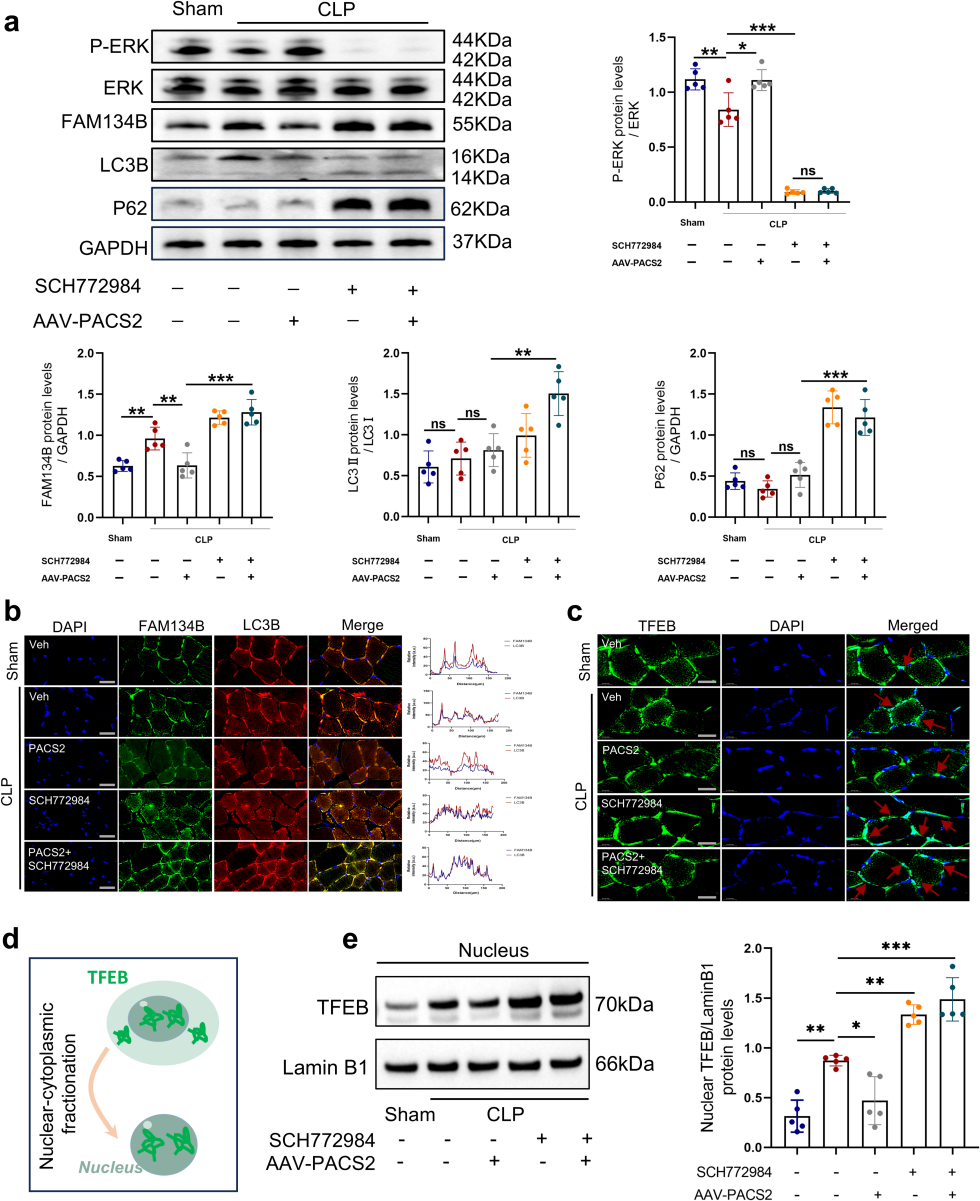
ERK activation reduces the transcriptional levels of FAM134B in the skeletal muscles of septic mice by inhibiting the nuclear translocation of TFEB. Mice were divided into five groups based on different treatments, Sham, CLP, CLP+AAV‐PACS2, CLP+SCH772984 and CLP+AAV‐PACS2+SCH772984 group (*n* = 5). (a) Western blot analysis of p‐ERK, ERK, FAM134B, LC3B, p62 protein levels and in TA muscle from each group. ERK phosphorylation is expressed as the p‐ERK/ERK ratio to verify the inhibitory effect of SCH772984. FAM134B and p62 were normalized to GAPDH; LC3B is expressed as the LC3‐II/I ratio. (b) Immunofluorescence analysis of TA muscle. Blue fluorescence represents DAPI; green fluorescence represents FAM134B; red fluorescence represents LC3B. Merged images show co‐localization of FAM134B and LC3B, indicating ER‐phagy activity (*n* = 5). (c) Immunofluorescence analysis of TA muscle, green fluorescence represents TFEB; blue fluorescence represents DAPI. Merged images show co‐localization of TFEB and DAPI, indicating TFEB nuclear translocation. (d) Schematic diagram of TFEB nuclear‐cytoplasmic fractionation. (e) Representative Western blots and quantitative analysis of TFEB protein levels in nuclear fractions of each group, with results normalized to levels of Lamin B1. Data are means ± SD *P*‐value determined by ordinary one‐way ANOVA. *ns*, not significant; *p < 0.05; **p < 0.01; ***p < 0.001.

To elucidate the role of ERK–MAPK signalling in SIM, mice received SCH772984 (a specific ERK inhibitor), and FAM134B expression was assessed. Western blot confirmed effective ERK inhibition (p‐ERK/ERK ratio decreased by approximately 88% in CLP+SCH772984 vs. CLP). SCH772984 reversed PACS2‐mediated FAM134B suppression: while PACS2 overexpression reduced FAM134B in CLP+AAV‐PACS2 mice, SCH772984 co‐treatment (CLP+AAV‐PACS2+SCH772984) restored FAM134B levels. Similarly, LC3 and p62 expressions increased with SCH772984 treatment (Figure 7a). Immunofluorescence confirmed that PACS2 overexpression decreased FAM134B and LC3B co‐localization, which was reversed by SCH772984 (Figures 7b and S2a), indicating that PACS2 suppresses ER‐phagy via ERK–MAPK activation.

Considering that p‐ERK cannot directly regulate FAM134B, we investigated downstream transcriptional mechanisms. The ERK–MAPK pathway modulates multiple transcription factors, among which TFEB serves as a master regulator bridging stress signalling to autophagy‐lysosomal gene expression. Upon activation, TFEB translocates into the nucleus to induce transcription of autophagy genes including FAM134B [20]. We therefore examined TFEB nuclear translocation in septic skeletal muscle.

Immunofluorescence analysis revealed that CLP induced TFEB nuclear translocation, which was significantly suppressed by PACS2 overexpression (approximately 36% reduction) but restored by SCH772984 co‐treatment (Figures 7c and S2b). To further validate the differential nuclear localization of TFEB, we performed cytoplasmic and nuclear fractionation (Figures 7d and S2c). Quantitative analysis of nuclear fractions confirmed that PACS2 overexpression markedly decreased nuclear TFEB levels, whereas SCH772984 co‐treatment restored TFEB nuclear accumulation (Figure 7e). CMAP analysis showed that PACS2 overexpression ameliorated sepsis‐induced neuromuscular dysfunction, but SCH772984 abolished this protection (reduced amplitude, prolonged latency) (Figure S2d). Histologically, PACS2 overexpression prevented CLP‐induced myofiber atrophy, whereas SCH772984 negated this effect, decreasing normalized myofiber CSA (Figure S2e). Consistently, MuRF1 and Atrogin‐1 levels increased with ERK inhibition (Figure S2f). These data demonstrate that PACS2 alleviates CLP‐induced muscle atrophy by activating ERK–MAPK, thereby reducing FAM134B expression and ER‐phagy.

## 4 Discussion

The ER and mitochondria interact to form a specialized structure called the mitochondria‐associated ER membranes, which play a critical role in the initiation and regulation of ER‐phagy and influence cell survival and death [21]. Studies have demonstrated that disruption of MAM structure leads to various muscle diseases, including skeletal muscle insulin resistance [22] and SEPN1‐related myopathy [23]. In this study, we found that sepsis decreased PACS2 protein expression, which disrupted MAM structural integrity, promoted FAM134B‐mediated ER‐phagy and ultimately contributed to skeletal muscle atrophy in mice.

PACS2, a multifunctional sorting protein localized to MAM, regulates ER and mitochondrial membrane dynamics, and its downregulation leads to fragmentation of both organelles, thereby impairing MAM structure and function [6]. Our results demonstrated that sepsis induced MAM structural damage and ER‐phagy activation in mouse skeletal muscle, both of which were associated with decreased PACS2 expression. Given the intimate physical and functional coupling between the ER and mitochondria, MAM‐mediated ER‐phagy may reciprocally affect MAM structure, thereby compromising mitochondrial function. Previous research has demonstrated that FAM134B regulates ER‐phagy to mitigate hippocampal neuronal apoptosis in acquired epilepsy, leading to MAM disruption and decreased mitochondrial calcium uptake [24]. Furthermore, FAM134B directly interacts with the mitochondrial inner membrane protein OPA1, recruiting ER membranes to form autophagic structures that engulf mitochondria, thereby promoting mitophagy [25]. Therefore, whether FAM134B‐mediated ER‐phagy in SIM reciprocally affects MAM structure and mitochondrial function, consequently exacerbating or ameliorating skeletal muscle atrophy, remains to be further investigated.

Additionally, Zheng et al. [26] found that ER stress promotes skeletal muscle atrophy in septic mice by activating STAT3 and Smad3. However, the involvement of ER‐phagy in SIM pathogenesis has not been previously reported. In the present study, we found that upregulated FAM134B expression in skeletal muscle of septic mice mediated ER‐phagy hyperactivation, leading to muscle atrophy. Song et al. [27] comprehensively reviewed the interplay between ER stress and ER‐phagy, proposing that ER stress serves as an upstream mechanism for ER‐phagy. Conversely, Jin et al. [28] found that enhanced ER‐phagy alleviated soleus muscle injury induced by acute exercise in rats through attenuation of ER stress. Moreover, Yang et al. [29] demonstrated that FAM134B activated ER‐phagy, induced ER stress and exacerbated cisplatin‐induced ototoxicity. Collectively, these findings suggest that while ER‐phagy and ER stress are interconnected, they may also independently contribute to muscle injury.

MAPKs are pivotal signal transduction enzymes regulating diverse aspects of mammalian physiology, including cellular ageing, stress responses and energy metabolism. Our results demonstrated that PACS2 overexpression activated the ERK–MAPK signalling pathway in the skeletal muscle of septic mice, without affecting p‐P38 or p‐JNK levels. Given that p‐ERK cannot directly regulate FAM134B expression, we investigated the underlying mechanisms. Recent studies have shown that ERK activation reduces autophagy levels by inhibiting TFEB nuclear translocation, thereby exacerbating the progression of fatty liver disease [30], Alzheimer's disease [31], and oesophageal cancer [32, 33]. Furthermore, research has demonstrated that the transcription factor TFEB directly binds to the CLEAR consensus sequence in the FAM134B promoter to promote FAM134B transcription [34]. We therefore hypothesized that PACS2 suppresses ER‐phagy through ERK–MAPK‐mediated inhibition of TFEB nuclear translocation, consequently attenuating FAM134B transcriptional activity. Our experimental findings support this hypothesis, as treatment with the ERK inhibitor SCH772984 promoted TFEB nuclear translocation and reversed the suppressive effect of PACS2 overexpression on TFEB‐mediated transcription.

Despite these findings, several limitations should be acknowledged. First, direct intramuscular AAV injection precluded accurate ex vivo functional assessment due to localized tissue disruption. Future studies will employ systemic AAV delivery via tail vein injection to address this limitation. Second, while FAM134B phosphorylation and oligomerization are known to regulate ER‐phagy [35], their roles in SIM pathogenesis remain to be investigated. Similarly, PACS2 contains multiple phosphorylation sites involved in cellular metabolism and MAM function [36, 37], yet whether sepsis alters PACS2 phosphorylation and which sites are modified remain intriguing and open questions. Notably, AKT‐mediated PACS2 phosphorylation has been shown to promote its interaction with 14‐3‐3 proteins, facilitating cell survival [38]. Whether this mechanism activates ERK–MAPK signalling and consequently improves skeletal muscle function in septic mice requires further study.

## 5 Conclusion

**FIGURE 8:**
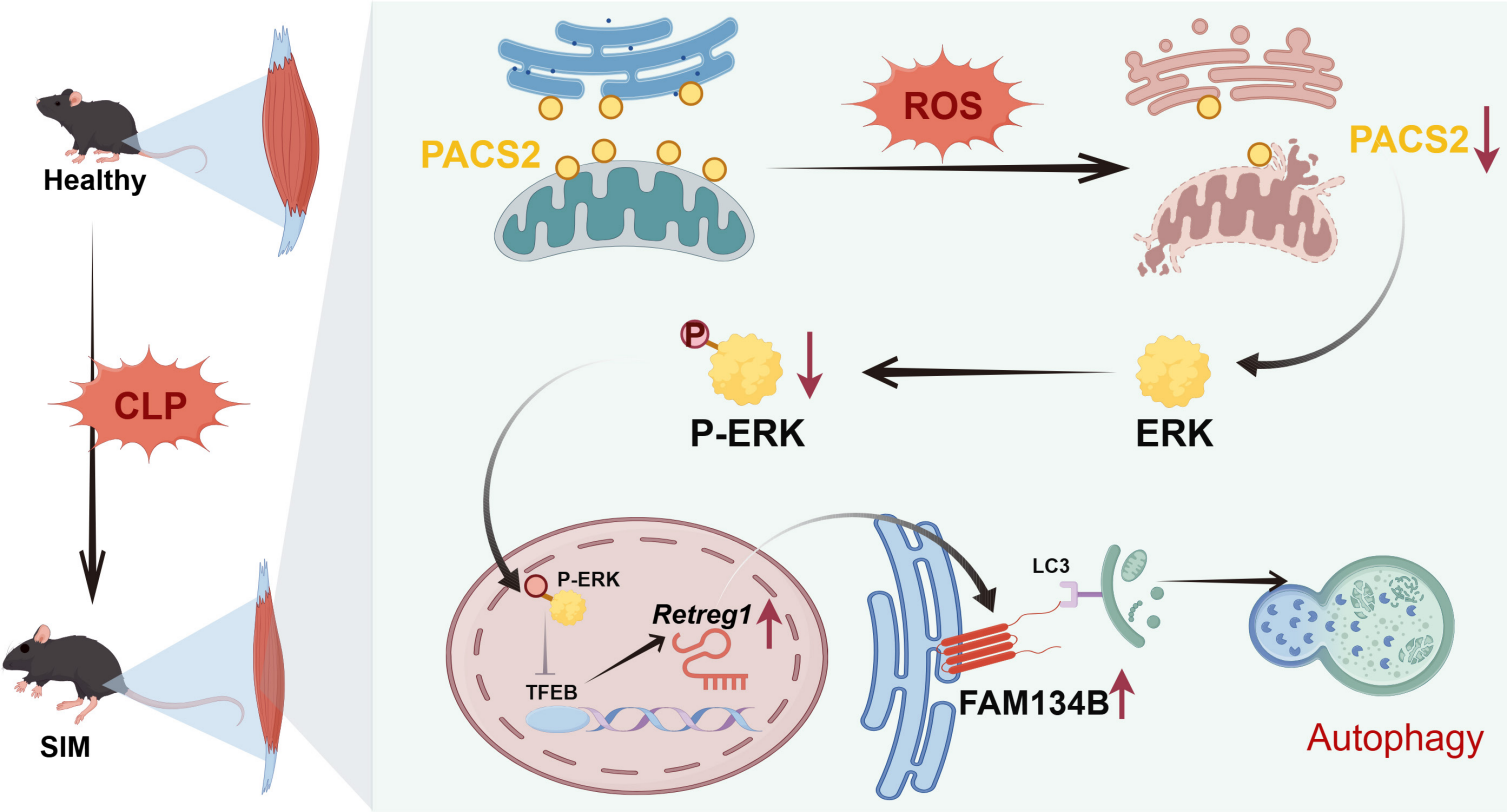
Schematic diagram of PACS2 downregulation‐induced skeletal muscle atrophy in septic mice. Sepsis reduces PACS2 expression in mitochondria‐associated endoplasmic reticulum membranes of skeletal muscle, leading to decreased ERK phosphorylation. This attenuates the inhibitory effect of p‐ERK on TFEB nuclear translocation, thereby enhancing TFEB‐mediated transcriptional activation of FAM134B. Consequently, upregulated FAM134B promotes excessive ER‐phagy, ultimately resulting in skeletal muscle atrophy.

In conclusion, our current experimental findings suggested that SIM is associated with excessive ER‐phagy mediated by increased FAM134B expression, which is driven by the decrease of PACS2 expression and the subsequent disruption of MAM integrity. Overexpression of PACS2, through the activation of the ERK–MAPK signalling pathway, inhibited TFEB nuclear translocation and reduced the transcriptional levels of FAM134B, thereby reversing this process (Figure 8).

## Funding

This work was supported by the Sichuan Science and Technology Program (2022YFS0632) and the Sichuan Provincial Medical Research Project (no. S23046).

## Ethics Statement

All experimental designs and protocols involving animals were approved by the Institutional Animals Ethics Committee at Southwest Medical University, China (approval no. SWMU20240075; Luzhou, China).

## Conflicts of Interest

The authors declare no conflicts of interest.

## Supporting information


**Figure S1:** Sepsis decreases PACS2 expression in skeletal muscle of mice.
**Figure S2:** ERK inhibition attenuates the protective effects of PACS2 overexpression on skeletal muscle in septic mice.

## Data Availability

The data are available from the corresponding author on reasonable request.
