# Supplementary material for: PACS2 Alleviates Sepsis‐Induced Myopathy by Activating ERK–MAPK Signalling Pathway to Suppress ER‐Phagy
Source: J Cachexia Sarcopenia Muscle. 2026 May 8;17(3):e70308. doi: 10.1002/jcsm.70308 (PMC13156248; doi:10.1002/jcsm.70308)
Supplement: Supplementary file 1 — Figure S1: Sepsis decreases PACS2 expression in skeletal muscle of mice. Figure S2: ERK inhibition attenuates the protective effects of PACS2 overexpression on skeletal muscle in septic mice. [file JCSM-17-e70308-s001.docx]

**Supporting Information**

**1. Methods**

***1.1 Animal Serum IL-6, TNF-a, and IL-1β Measurements***

Blood samples were collected via cardiac puncture at designated time points following CLP modeling and centrifuged at 1000 × g for 15 min at 4°C. Serum concentrations of IL-6, TNF-α, and IL-1β were measured using ELISA kits (MEIMIAN, China) according to the manufacturer's instructions.

***1.2 Skeletal Muscle Weight Measurement***

Mice from the CLP and sham groups were humanely euthanized under anesthesia. Bilateral tibialis anterior (TA) and gastrocnemius (GAS) muscles were carefully dissected, blotted dry with filter paper, and immediately weighed.

***1.3 Compound Muscle Action Potential Measurement***

Mice were anaesthetized and placed on a heating pad, and then electromyography measurements were performed before euthanasia. Measurements were conducted using an RM6240 multichannel physiological signal acquisition and processing system. Compound muscle action potential (CMAP) measurements were performed to assess hindlimb nerve functionality. For the GAS muscle, the anode and cathode subdermal electrodes were positioned proximal to the sciatic notch, 0.5 cm apart from each other, while the recording electrode was placed at the site of maximum GAS muscle diameter. A reference electrode was placed at the ipsilateral ankle and the ground electrode on the contralateral side. Latency was measured from the stimulation to the start of CMAP response, and amplitude was determined from the maximum negative peak to the maximum positive peak of the bidirectional wave. Latency was used to evaluate the nerve conduction time, and amplitude reflected the number of depolarizing muscle fibers. All measurements were performed in triplicate and analyzed as average values.

***1.4 Grip Strength Test***

After acclimatization to the grip strength meter, the peak forelimb grip strength of mice was assessed on the day before CLP surgery, as well as at 24 and 96 hours post-surgery. Each mouse was placed on the metal grid and allowed to grasp it firmly with its forepaws. The mouse was then pulled backwards along the horizontal plane until it released the metal grid. Grip strength was measured three times with rest intervals between trials. The mean value of the three measurements was used for analysis and normalized to body weight.

***1.5 Immunofluorescence Staining***

Immunofluorescence staining was conducted to detect markers of ER-phagy and MAM structures and localize TFEB. Slides were deparaffinized in xylene, rehydrated through graded ethanol, and subjected to antigen retrieval. After cooling to room temperature, sections were blocked with 5% bovine serum albumin containing 0.5% Triton X-100 for 1 h at room temperature. Slices were then incubated overnight at 4 °C with primary antibodies: anti-FAM134B (Proteintech, 21537-1-AP, 1:50), anti-LC3β (Santa Cruz, SC-271625, 1:50), anti-IP3R (Santa Cruz, SC-377518, 1:50), anti-VDAC1 (Abcam, ab14734, 1:50), and anti-TFEB (Proteintech, 13372-1-AP, 1:50). Following extensive washing with PBS, sections were incubated with appropriate fluorescent secondary antibodies for 1 h at room temperature. Nuclei were counterstained with DAPI. Images were acquired using a confocal laser scanning microscope (LSM880, Zeiss), and fluorescence intensity was quantified using ImageJ software.

***1.6 Transmission Electron Microscopy***

TA muscles were dissected and immediately fixed in 2.5% glutaraldehyde in 0.1 M phosphate buffer (pH 7.4) for 2 h at 4°C, followed by post-fixation in 1% osmium tetroxide for 1 h at room temperature. Samples were then washed three times with phosphate buffer, dehydrated through a graded series of ethanol, infiltrated and embedded in epoxy resin. Ultrathin sections (60-80 nm) were prepared using an ultramicrotome (EM UC7, Leica) and mounted on copper grids. Sections were counterstained with uranyl acetate and lead citrate. Ultrastructural images of TA muscle were acquired using a transmission electron microscope (Thermo Fisher Scientific, Talos F200C).

***1.7 RNA-Sequencing***

TA muscles from Sham-96 h and CLP-96 h groups, or AAV-CON-CLP and AAV-PACS2-CLP groups, were collected and frozen for subsequent RNA extraction. Stranded RNA‐Seq was performed on 500 ng of total RNA from each sample. RNA quality was checked using Bioanalyzer instrument (Agilent, USA), quantified with ND‐2000 (NanoDrop Technologies), and then subjected to cDNA library construction. Analysis of differentially expressed genes was performed on the Genomatix Genome Analyzer using the DESeq2 software package (two-tailed Wald’s test). Functional enrichment and pathway analysis of differentially expressed genes were performed using Enrichr and Reactome software.

***1.8 RNA Extraction and Quantitative RT–PCR (qRT-PCR)***

Total RNA was extracted from TA muscle tissues using TRIzol reagent (TIANGEN, DP424) according to the manufacturer's instructions. Single-stranded cDNA was synthesized using the SuperScript IV VILO Master Mix (Thermo Fisher Scientific, 11755-050). qRT-PCR was performed using TB Green Premix Ex Taq II (Takara, RR820A) on a QuantStudio 3 Real-Time PCR System. The primer sequences used were as follows: PACS2 forward, 5′-ACAGCTAAGCACCAACAGAACAT-3′, PACS2 reverse, 5′-AAGAACTTGACATCGCTGCATTC-3′; GAPDH forward, 5′-GGTTGTCTCCTGCGACTTCA-3′, GAPDH reverse, 5′-CCCTGTTGCTGTAGCCGTAT-3′. Relative mRNA expression levels were normalized to GAPDH and calculated using the 2^^-ΔΔ^Ct method.

**2. Results**

**Figure. S1**

**
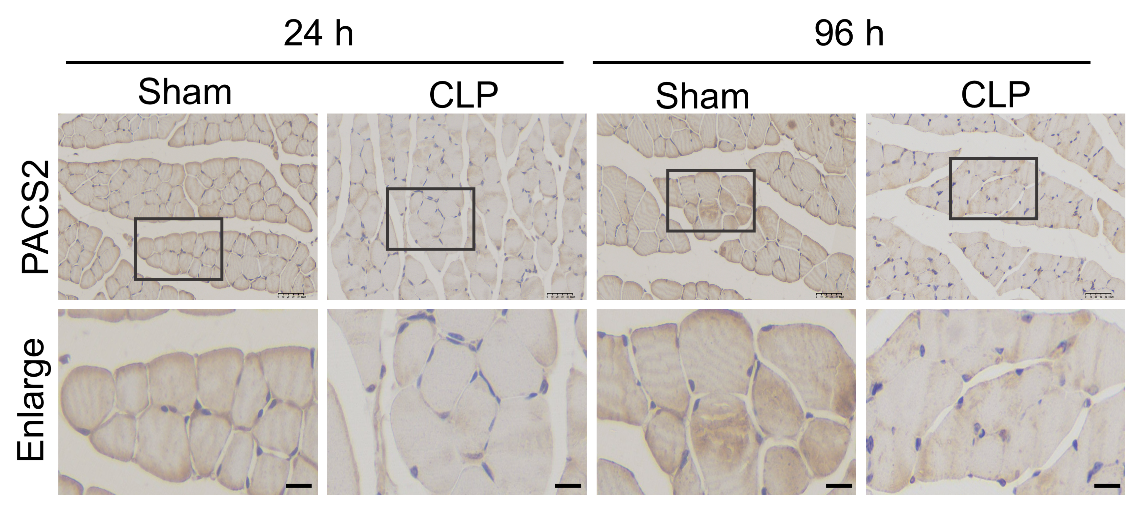
**

**Supplementary Figure S1. Sepsis Decreases PACS2 Expression in Skeletal Muscle of Mice.**

Sepsis mice model was established by CLP. Sham-24 h and sham-96 h groups were only sham-operated, CLP-24 h and CLP-96 h groups were detected at 24 h and 96 h after operation. Representative immunohistochemical staining images of PACS2 in the TA muscle from each group. Sepsis significantly decreased PACS2 expression in mice skeletal muscle compared to sham controls.

**Figure. S2**


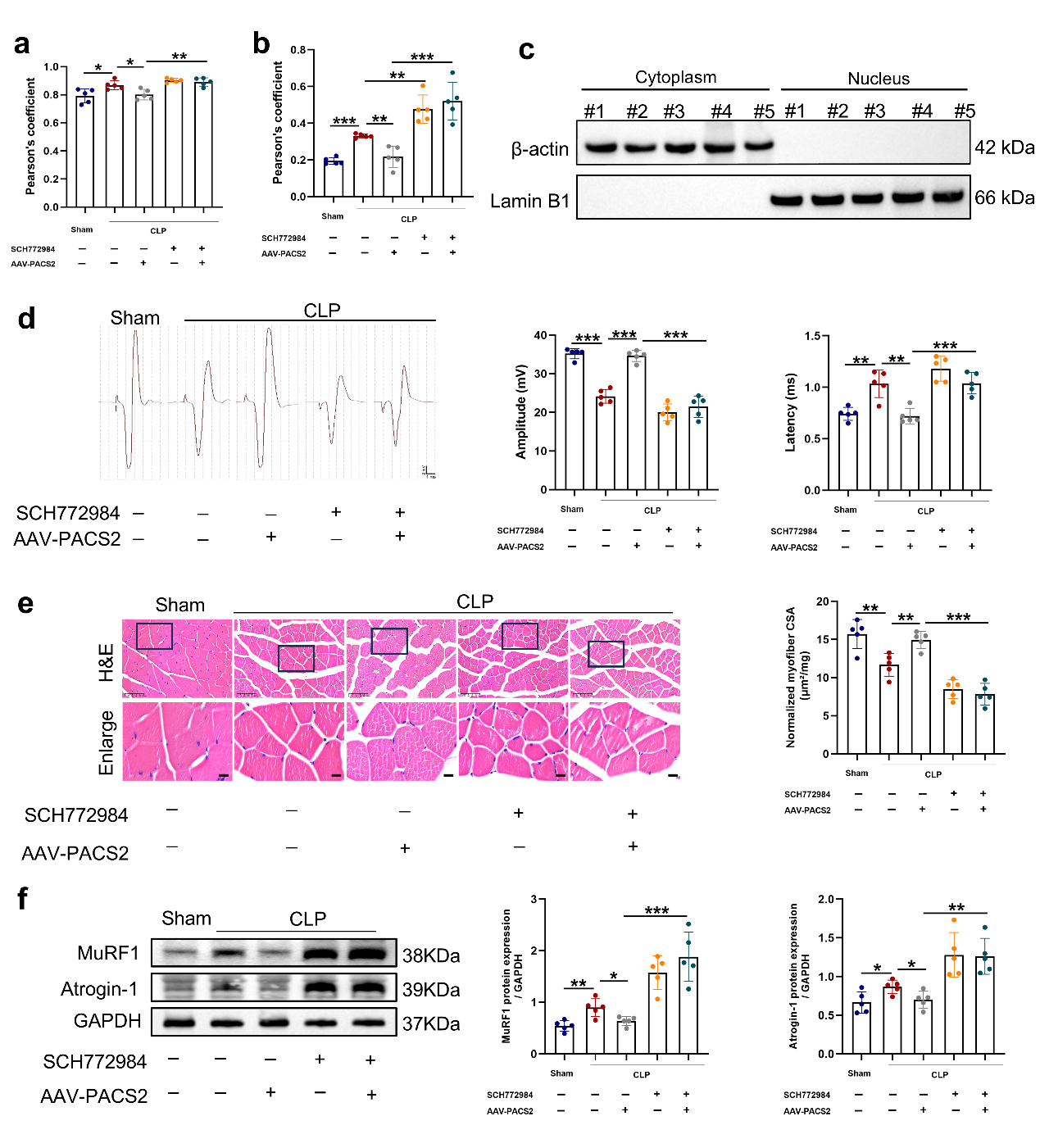


**Supplementary Figure S2. ERK Inhibition Attenuates the Protective Effects of PACS2 Overexpression on Skeletal Muscle in Septic Mice.**

Mice were divided into five groups based on different treatments, Sham, CLP, CLP+AAV-PACS2, CLP+SCH772984 and CLP+AAV-PACS2+SCH772984 group (*n*=5). **(a)**: Quantitative analysis of Pearson’s correlation coefficient (PCC) for FAM134B and LC3B co-localization. **(b)**: Quantitative analysis of PCC for TFEB and DAPI co-localization. **(c)**: Western blot analysis of nuclear marker (Lamin B1) and cytoplasmic marker (β-actin) in nuclear and cytoplasmic fractions isolated to verify fractionation efficiency. **(d)**: Skeletal muscle function was measured by measuring sciatic nerve GAS muscle CMAP. Red arrow indicates single stimulation. Amplitude was determined from the maximum negative peak to the maximum positive peak of the biphasic wave, which indicates the number of depolarizing muscle fibers (*n*=5). Latency was measured from stimulation onset to CMAP response initiation, which reflects nerve-to-muscle signal conduction time (*n*=5). (e): Quantification of GAS muscle fiber cross-sectional areas (CSA) from H&E-stained sections (*n*=5). Representative images at 20× magnification are shown (scale bar: 100 μm). CSA values were normalized to muscle wet weight and expressed as μm²/mg. (**f)**: Western blot analysis of the dynamic changes of MuRF1 and Atrogin-1 protein levels in TA muscle of each group, results normalized to levels of GAPDH. Data are means ± SD. *P*-value determined by ordinary one-way ANOVA. **p* < 0.05; ***p* < 0.01; ****p* < 0.001.
